# Comparative analysis of *Listeria monocytogenes* plasmid transcriptomes reveals common and plasmid‐specific gene expression patterns and high expression of noncoding RNAs

**DOI:** 10.1002/mbo3.1315

**Published:** 2022-09-19

**Authors:** Justin M. Anast, Andrea J. Etter, Stephan Schmitz‐Esser

**Affiliations:** ^1^ Department of Animal Science Iowa State University Ames Iowa USA; ^2^ Interdepartmental Microbiology Graduate Program Iowa State University Ames Iowa USA; ^3^ Department of Nutrition and Food Sciences The University of Vermont Burlington Vermont USA

**Keywords:** *Listeria monocytogenes*, noncoding RNA, plasmid, transcriptome

## Abstract

Recent research demonstrated that some *Listeria monocytogenes* plasmids contribute to stress survival. However, only a few studies have analyzed gene expression patterns of *L. monocytogenes* plasmids. In this study, we identified four previously published stress‐response‐associated transcriptomic data sets which studied plasmid‐harboring *L. monocytogenes* strains but did not include an analysis of the plasmid transcriptomes. The four transcriptome data sets encompass three distinct plasmids from three different *L. monocytogenes* strains. Differential gene expression analysis of these plasmids revealed that the number of differentially expressed (DE) *L. monocytogenes* plasmid genes ranged from 30 to 45 with log_2_ fold changes of −2.2 to 6.8, depending on the plasmid. Genes often found to be DE included the cadmium resistance genes *cadA* and *cadC*, a gene encoding a putative NADH peroxidase, the putative ultraviolet resistance gene *uvrX*, and several uncharacterized noncoding RNAs (ncRNAs). Plasmid‐encoded ncRNAs were consistently among the highest expressed genes. In addition, one of the data sets utilized the same experimental conditions for two different strains harboring distinct plasmids. We found that the gene expression patterns of these two *L. monocytogenes* plasmids were highly divergent despite the identical treatments. These data suggest plasmid‐specific gene expression responses to environmental stimuli and differential plasmid regulation mechanisms between *L. monocytogenes* strains. Our findings further our understanding of the dynamic expression of *L. monocytogenes* plasmid‐encoded genes in diverse environmental conditions and highlight the need to expand the study of *L. monocytogenes* plasmid genes' functions.

## Graphical abstract

This study performed gene expression analysis of three *Listeria monocytogenes* plasmids from three different *L. monocytogenes* strains. These findings further our understanding of the dynamic expression of *L. monocytogenes* plasmid‐encoded genes in diverse environmental conditions and highlight the need to expand the study of *L. monocytogenes* plasmid gene function

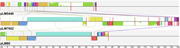

## 1 INTRODUCTION

*Listeria monocytogenes* is a Gram‐positive facultative anaerobe and the causative agent of listeriosis, a rare but highly fatal foodborne illness (Buchanan et al., 2017; Schlech, 2019). *L. monocytogenes* employs a complex array of regulatory and stress response mechanisms to transition from a saprophytic to a pathogenic lifecycle (Freitag et al., 2009; Gaballa et al., 2019; Johansson & Freitag, 2019). After *L. monocytogenes* reaches the gastrointestinal tract lumen, systemic infection occurs when the pathogen translocates across the epithelium and disseminates to peripheral organs through the circulatory and lymphatic systems (Radoshevich & Cossart, 2018). *L. monocytogenes* is of considerable concern for food producers due to its ability to survive over extended periods in food production environments (FPEs). Indeed, this phenomenon of persistence, where the same strains of *L. monocytogenes* are repeatedly isolated from the same FPE over multiple months or years, has been shown to last up to several decades despite routine, rigorous sanitation of facilities (Carpentier & Cerf, 2011; Etter et al., 2017; Ferreira et al., 2014; Hammons et al., 2017). While not fully understood, this persistence is in part due to several molecular mechanisms that provide protection against low pH (Cotter et al., 2000; Feehily et al., 2014; Lund et al., 2014), oxidative stress (Harter et al., 2017), disinfectants (Elhanafi et al., 2010; Jiang et al., 2020; Muller et al., 2013), high osmotic pressure (Bucur et al., 2018), temperatures as low as −0.4°C (Chan & Wiedmann, 2009), and ultraviolet light (Kim et al., 2006).

In addition to well‐characterized chromosomally encoded stress response systems, some *L. monocytogenes* plasmids increase tolerance to various stress conditions, including elevated temperature, ultraviolet light, salt concentrations, lactic acid, and disinfectants (Anast & Schmitz‐Esser, 2021; Elhanafi et al., 2010; Naditz et al., 2019; Pontinen et al., 2017). On average, 47%−54% of *L. monocytogenes* strains harbor a putative plasmid, although plasmid prevalence between different sequence types is highly variable (Chmielowska et al., 2021; Schmitz‐Esser et al., 2021). *L. monocytogenes* plasmids are highly conserved across strains in a modular fashion in which certain regions possess high degrees of sequence similarity to regions on other plasmids (Kuenne et al., 2010; Schmitz‐Esser et al., 2021). However, while modules themselves can be highly conserved, the number and identity of modules on a given *L. monocytogenes* plasmid can be highly variable (Kuenne et al., 2010; Schmitz‐Esser et al., 2021). Many of these modules contain genes predicted to be involved in stress response; recent comparative genetic analysis has revealed that *L. monocytogenes* plasmids encode several putative stress response genes (Hingston et al., 2019; Kuenne et al., 2010; Naditz et al., 2019; Pontinen et al., 2017; Schmitz‐Esser et al., 2021). Some common stress‐associated proteins encoded by *L. monocytogenes* plasmid genes include (1) TMR, a triphenylmethane reductase that increased tolerance to antimicrobial dyes (Dutta et al., 2014), (2) the cadmium efflux proteins CadA1 (from the transposon Tn*5422*) and CadA2 (Lebrun et al., 1994; Parsons et al., 2018), (3) a multicopper oxidase (MCO) involved in heavy metal resistance in *Staphylococcus aureus* (Sitthisak et al., 2005), (4) quaternary ammonia compound resistance proteins BcrABC (Elhanafi et al., 2010), (5) a putative NADH peroxidase (Hingston et al., 2019), and (6) the heat shock protein ClpL (Pontinen et al., 2017). Additionally, plasmid‐borne noncoding RNAs (ncRNAs) are implicated in the *L. monocytogenes* stress response, evidenced by the upregulation of a putative nickel−cobalt (NiCo) riboswitch during lactic acid stress exposure (Cortes et al., 2020). Indeed, other putative ncRNAs are harbored on *L. monocytogenes* plasmids (Kuenne et al., 2010). While several studies highlight the importance of plasmids during the saprophytic stress‐response of *L. monocytogenes* (Anast & Schmitz‐Esser, 2021; Elhanafi et al., 2010; Naditz et al., 2019; Pontinen et al., 2017), currently, only one study has demonstrated a relationship between an *L. monocytogenes* plasmid and virulence (Den Bakker et al., 2012).

Whole transcriptome sequencing is widely used in *L. monocytogenes* research to elucidate genes of interest involved in virulence, saprophytic stress survival, and gene expression regulatory mechanisms (Anast & Schmitz‐Esser, 2020; Assisi et al., 2021; Behrens et al., 2014; Cortes et al., 2020; Guariglia‐Oropeza et al., 2018; Hingston, Chen, Allen, et al., 2017; Kragh & Truelstrup Hansen, 2019; Marinho et al., 2019; Mraheil et al., 2011; Soni et al., 2011; Tang et al., 2015; Vivant et al., 2017; Wehner et al., 2014; Wurtzel et al., 2012). However, transcriptome sequencing studies that include an analysis of plasmid gene expression in *L. monocytogenes* are limited in number (Anast & Schmitz‐Esser, 2020; Cortes et al., 2020; Kragh & Truelstrup Hansen, 2019). Several studies that conducted gene expression analysis on plasmid‐harboring *L. monocytogenes* strains did not evaluate plasmid gene expression due to methodological constraints (Assisi et al., 2021; Guariglia‐Oropeza et al., 2018; Tang et al., 2015). In these studies, the transcriptome sequencing reads were mapped to a reference *L. monocytogenes* genome different than the strain used for the gene expression experiments. These reference strains (*L. monocytogenes* EDG‐e and 10403S) do not contain a plasmid, and as a result, all plasmid reads were discarded in the read mapping. We thus sought to utilize these available, novel transcriptomic data to improve our understanding of the role of *L. monocytogenes* plasmids in different conditions. These data sets cover three genetically distinct plasmids: the well‐studied *L. monocytogenes* plasmid pLM80 and two novel putative plasmids, pLM5446 and pLM7802, from the whole‐genome shotgun sequences of *L. monocytogenes* isolates FSL R8‐5446 (CU‐11‐320) and FSL R8‐7802 (CU‐259‐322), respectively.

## 2 METHODS

### 2.1 Review of the literature

This study aimed to improve our understanding of *L. monocytogenes* plasmid gene expression based on existing published data. Therefore, we searched NCBI PubMed for studies based on the following criteria: (1) transcriptome sequencing must have been performed on an *L. monocytogenes* strain; (2) the *L. monocytogenes* strain used in the transcriptomics experiment must have a sequenced and assembled genome; (3) the strain must harbor a putative plasmid; (4) the study must not have reported plasmid gene expression data. In strains where the existence of a plasmid was unknown, the genomes were subjected to protein BLAST (BLASTp) searches (method described below) with known *L. monocytogenes* plasmid replication protein RepA protein sequences as a query, and annotations from significant hits of the query sequences were manually reviewed for known *L. monocytogenes* plasmid proteins.

After a detailed literature search, we found three studies that fulfilled all criteria (Tables 1 and 2) (Assisi et al., 2021; Guariglia‐Oropeza et al., 2018; Tang et al., 2015). Two studies were excluded because they already performed detailed characterization of plasmid gene expression of the plasmids pLM6179 and pLMR479a (Anast & Schmitz‐Esser, 2020; Cortes et al., 2020). In addition, another study (Kragh & Truelstrup Hansen, 2019) was excluded because no DE genes were detected on plasmid pLM5578. Two of the transcriptomes reanalyzed here for plasmid gene expression (Guariglia‐Oropeza et al., 2018; Tang et al., 2015) utilized the *L. monocytogenes* strain H7858, a strain that was isolated from hot dogs during a listeriosis outbreak in 1998 (Centers for Disease Control and Prevention, 1998; Nelson et al., 2004). H7858 is a well‐characterized *L. monocytogenes* strain and harbors the plasmid pLM80, which was assembled into two contigs (accession NZ_AADR01000010; NZ_AADR01000058). In these two studies, transcriptome reads were mapped to a pseudochromosome generated by aligning H7858 contigs to the chromosome of *L. monocytogenes* EGD‐e. However, because EGD‐e does not possess a plasmid, the H7858 plasmid contigs were not aligned and thus not incorporated into the H7858 pseudochromosome. As a result, any reads originating from pLM80 were not mapped and analyzed in either study.

In the first of these studies, Tang et al. (2015) compared the transcriptomes of H7858 grown at 7°C on vacuum‐packed cold‐smoked salmon (CSS) and in a modified brain heart infusion broth (MBHIB) (Tables 1 and 2). MBHIB is distinct from typical brain heart infusion broth (BHI) as the salt concentration was modified to 4.65%, and the pH was adjusted to 6.1 to better mimic conditions found on CSS. In the second study, Guariglia‐Oropeza et al. (2018), cultured H7858 in BHI broth containing 1.1% porcine bile and compared gene expression against cells grown in standard BHI at pH 5.5 as a control (Tables 1 and 2). All incubations conducted by Guariglia‐Oropeza et al. (2018) were performed at 37°C.

The final study chosen for this analysis was conducted by Assisi et al. (2021). In their investigation, the authors sequenced and assembled the genomes of 21 *L. monocytogenes* isolates derived from the deli retail environment, and four of these isolates were chosen for subsequent gene expression analysis comparing growth in biofilm versus planktonic broth growth (Tables 1 and 2). Similar to the other studies, the authors mapped reads to the genome of *L. monocytogenes* 10403S, a strain that lacks a plasmid, thus overlooking potential differences in plasmid gene expression between conditions.

**Table 1:** Strains and sequencing information from published transcriptome sequencing data sets reanalyzed in this study for plasmid gene expression

	Tang et al. (2015)	Guariglia‐Oropeza et al. (2018)	Assisi et al. (2021)
*Listeria monocytogenes* strain(s) (MLST sequence type)	H7858 (ST6)	H7858 (ST6)	FSL R8‐5446 (ST5)
			FSL R8‐7802 (ST6)
Plasmid(s) (assembly size kb)	pLM80 (82.2)	pLM80 (82.2)	pLM5446 (73.1)
			pLM7802 (135.9)
Genome sequencing accession number	AADR00000000	AADR00000000	JAGYVF000000000
			JAGYVE000000000
Transcriptome sequencing BioProject	PRJNA270808	PRJNA401799	PRJNA736746
Transcriptome sequencing platform, read length	Illumina HiSeq. 2000 SE^a^, 100 bp	Illumina HiSeq. 2500 SE^a^, 100 bp	Illumina HiSeq. 2500 SE^a^, 100 bp

^a^ SE denotes that sequence reads are single‐end.

**Table 2:** Experimental design and conditions from gene expression studies reanalyzed in this study

	Tang et al., (2015)	Guariglia‐Oropeza et al. (2018)	Assisi et al. (2021)
Purpose of study	To compare the *L. monocytogenes* transcriptome in CSS against growth in broth	To better understand *L. monocytogenes* gene regulatory responses to bile stimulation	To identify differences in expression of *L. monocytogenes* stress/biofilm‐associated genes, which may be linked to persistence
Broth culture conditions	7‐day cultivation, 7°C, MBHIB, static, pH 6.1	10‐min exposure, 37°C, BHI with and without bile, static, pH 5.5	24 h cultivation, 20°C, BHI, 200 rpm
Biofilm culture conditions	7‐day cultivation, 7°C, CSS, pH 6.1	Condition not analyzed in the original study	72‐h cultivation, 20°C, stainless steel coupons submerged in BHI
Biologically independent replicates per condition	4	4	3

Abbreviations: BHI, brain heart infusion; CSS, cold‐smoked salmon; MBHIB, modified brain heart infusion broth.

### 2.2 Data acquisition

Raw sequence reads from Tang et al. (2015) were obtained from the NCBI Sequence Read Archive database, and reads from Guariglia‐Oropeza et al. (2018) and Assisi et al. (2021) were kindly provided by the authors. Detailed information about these data sets can be found in Table 1.

### 2.3 Identification of putative plasmid contigs

Identification of putative plasmid contigs was done using the methodology described by Schmitz‐Esser et al. (2021). Briefly, assembled *L. monocytogenes* genomes were first assessed for plasmid presence using *L. monocytogenes* RepA protein sequences as BLASTp queries. For this, the amino acid sequences representing a Group 2 RepA (pLM80, GenBank accession number WP_003726391) and a Group 1 RepA (pLM33, YP_003727990.1) were used as query sequences. Because *L. monocytogenes* plasmids are highly conserved (Korsak et al., 2019; Schmitz‐Esser et al., 2021), only hits with ≥90% amino acid sequence identity were considered for further analysis. After identifying which strains harbored RepA proteins in their assemblies, whole *L. monocytogenes* plasmid nucleotide sequences of pLM58 (GenBank accession number NZ_CP023753.1), pLIS1 (NZ_MH382833.1), pLMR479a (NZ_HG813248.1), pOB080183 (NZ_CP060527.1), and pLMN‐001A (CP006611.1) were used as nucleotide BLAST queries to identify which assembled contigs from each *L. monocytogenes* strain may contain plasmid sequences. These plasmids were selected because they represent large distinct and diverse *Listeria* plasmids (Schmitz‐Esser et al., 2021). We used several distinct *Listeria* plasmids as query sequences for the BlastN searches to maximize the likelihood of identifying plasmid contigs and to increase the reliability of identification of plasmid contigs. Again, because *L. monocytogenes* plasmids are well established to be highly conserved and modular (Chmielowska et al., 2021; Kuenne et al., 2010; Schmitz‐Esser et al., 2021), only contigs with matches of ≥95% nucleotide sequence similarity and at least a total alignment length of 500 bp were considered for further manual analysis of gene content.

### 2.4 Comparative genetic analysis

Existing gene annotations were verified where possible with NCBI BLASTp, Uniprot (UniProt Consortium, 2018), PATRIC (Brettin et al., 2015), eggNOG‐mapper (Cantalapiedra et al., 2021; Huerta‐Cepas et al., 2019), and Pfam webservers (Finn et al., 2016). The proteomes of plasmids pLM80, pLM5446, and pLM7802 were compared with the PATRIC proteome comparison tool (Davis et al., 2020). Only matches with at least 85% amino acid identity or greater were considered homologs between plasmids. The average nucleotide identities between pLM80, pLM5446, and pLM7802 were determined using the JSpeciesWS online server (https://jspecies.ribohost.com/jspeciesws/#Home) (Richter et al., 2016). Alignments of plasmids were generated using MAUVE (Darling et al., 2010). Putative ncRNAs were compared with and assessed for homologs in other bacteria using the nucleotide sequences as a query and searched against the RNA families (Rfam) database and server (https://rfam.xfam.org/). MCO amino acid sequences were aligned with MAFFT (Katoh & Toh, 2008); alignments were visualized using BOXSHADE.

### 2.5 Identification of homologs of previously described ncRNAs

The Infernal cmscan web server (https://www.ebi.ac.uk/Tools/rna/infernal_cmscan/rna/infernal_cmscan/) (Madeira et al., 2019) was used to analyze the plasmids for homologs of previously described ncRNAs. Genomic coordinates of the discovered putative ncRNA homologs were included in the annotated GenBank file by manually modifying the plasmid GenBank files for downstream analysis of the putative plasmid ncRNA gene expression.

### 2.6 Transcriptome analysis

Reads from the studies were mapped to either the *L. monocytogenes* H7858 (pLM80), FSL R8‐5446 (pLM5446), or FSL R8‐7802 (pLM7802) plasmid contigs using the Burrows−Wheeler aligner (H. Li & Durbin, 2010). For consistency, only the forward‐oriented reads of studies that used paired‐end sequencing were processed in this analysis. SAMtools (H. Li et al., 2009) was employed to examine and process SAM and BAM files. Read counts per predicted gene were calculated by ReadXplorer (Hilker et al., 2016) from the resulting BAM files, and statistical analysis was performed with the DESeq2 R package (Love et al., 2014) included in ReadXplorer to assess differential gene expression. DeSeq2 utilizes the Benjamini and Hochberg method for the correction of multiple‐testing to generate *Q* values (Benjamini & Hochberg, 1995); genes with *Q* values less than 0.05 were considered differentially expressed (DE). Transcripts per million (TPM) were calculated by ReadXplorer for normalization of gene expression by gene length and then by read depth. TPMs of the same gene from the same sample type (i.e., replicates of the same experimental condition and strain) were averaged in R to form consensus TPMs, and TPMs were ranked first (highest TPM value) to last (lowest TPM value) based on their average TPM value. Ranking of TPM expression level was used to qualitatively assess the transcriptional level of plasmid genes with respect to other genes on the same plasmid.

## 3 RESULTS AND DISCUSSION

**Figure 1:**
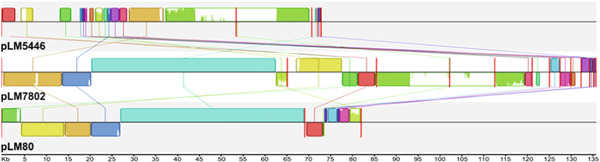
Mauve nucleotide alignment of pLM5446, pLM7802, and pLM80. Plasmid nucleotide sequences were aligned with MAUVE. Identically colored blocks denote homologous regions. Large red bars indicate contig boundaries. Bar heights within blocks of homologous regions correlate with the level of conservation shared between the plasmid sequences. The numerical scale is in kilobases.

Our analysis found that two of the four isolates used for transcriptome sequencing in the study conducted by Assisi et al. (2021), FSL R8‐5446 and FSL R8‐7802, harbored a novel, putative plasmid: pLM5446 and pLM7802, respectively (Table 3). The three plasmids in this study have been assembled in several contigs, with assembly sizes ranging from 73 to 135 kbp, and have a GC content ranging from 36.3% to 37.5% (Table 3). The aligned plasmid contigs showed high nucleotide sequence similarity between pLM80 and pLM7802 (Figure 1). Notably, the entire pLM80 plasmid was contained within the pLM7802 contigs, and overlapping sequences were virtually identical with >99.9% nucleotide identities. The pLM80‐like module of pLM7802 comprised 62% of the entire pLM7802 sequence. A low‐to‐moderate similarity was observed between pLM5446 and pLM80 and between pLM5446 and pLM7802 (Figure 1, Appendix Table A1, and Table S1: https://doi.org/10.6084/m9.figshare.20558421). The results of the genetic characterization of the plasmids are presented first by describing the genetic components of pLM80, pLM7802, and pLM5446. DE gene expression results are organized by plasmids pLM80, pLM7802, and pLM5446.

**Table 3:** General genetic features of plasmids used in this study

Plasmid	*L. monocytogenes* strain	No. of plasmid contigs	No. of plasmid CDS	Plasmid assembly size (bp)	Plasmid GC content (%)
pLM5446	FSL R8‐5446	4	74	73,132	36.3
pLM7802	FSL R8‐7802	12	154	135,980	36.8
pLM80	H7858	2	92	82,248	37.5

Abbreviation: CDS, predicted coding sequences.

### 3.1 Genetic analysis of pLM80

*L. monocytogenes* H7858 harbors the well‐studied plasmid pLM80, which is 82 kbp in size and possesses a Group 2 RepA protein (Locus_tag: LMOh7858_pLM80_0093) and several genes involved in stress response. These stress response genes include a cadmium resistance ATPase, *cadA* (Parsons et al., 2018), a benzalkonium chloride resistance cassette, *bcrABC* (Elhanafi et al., 2010), and the triphenylmethane reductase *tmr* which is known to increase tolerance toward crystal violet dye (Dutta et al., 2014). Additionally, pLM80 encodes a putative UV‐damage repair protein, *uvrX* (Locus_tag: LMOh7858_pLM80_0090), predicted to be a DNA polymerase that assists in the ultraviolet light stress‐response system (Kuenne et al., 2010). Kuenne et al. (2010) suggested that the common presence of *uvrX* on *L. monocytogenes* plasmids may indicate a mechanism that produces genetic deletions to increase the variation of the plasmids during DNA repair.

Kuenne et al. (2010) also identified a 40 kb region in pLM80 harboring a putative conjugation system containing predicted type IV secretion system proteins. pLM80 shares homology with the *Bacillus anthracis* plasmid pXO2, which shares a common backbone with the conjugative plasmid pAW63 of *Bacillus thuringiensis* (Van Der Auwera et al., 2005).

Two putative ncRNAs were predicted within pLM80 (Table 4), of which *rli28* has previously been identified by Kuenne et al. (2010). In several *L. monocytogenes* strains, *rli28* was also found on the chromosome where it is flanked by *lmo0470* and *lmo0471* homologs. In addition, *rli28* is a homolog (76% nucleotide identity and 86% query coverage) of the chromosomally encoded *rli50* (also known as *rli28*‐4) of *L. monocytogenes* EDG‐e (Rfam ID: RF01492). Previous research demonstrated that the chromosomally encoded *rli28* was induced in the intestine (Toledo‐Arana et al., 2009). When the transcriptomes of *L. monocytogenes* in standard culture broth were compared with samples cultivated in lagoon effluent (collected from a pig manure treatment processing unit), it was found that *rli28* and *rli50* were significantly downregulated in the lagoon effluent media (Vivant et al., 2017). In another study where *rli50* was deleted, *L. monocytogenes* virulence was attenuated in a murine macrophage model (Mraheil et al., 2011).

**Table 4:** Putative plasmid‐borne ncRNAs identified with Infernal

Plasmid	ncRNA homolog	Rfam family	Contig accession numbers, genetic coordinates
pLM80	*Listeria* sRNA Rli28	RF01492	NZ_AADR01000010, 27194‐27381
	RNA anti‐toxin A (RatA)	RF01776	NZ_AADR01000010, 27522‐27435
pLM7802	*Listeria* sRNA Rli28	RF01492	NODE_11_length_65401_cov_99.7279_ID_3, 20651‐20838
	NiCo riboswitch	RF02683	NODE_11_length_65401_cov_99.7279_ID_3, 2995‐2887
	RNA anti‐toxin A (RatA)	RF01776	NODE_11_length_65401_cov_99.7279_ID_3, 20979‐20892
	Fluoride riboswitch	RF01734	NODE_15_length_10341_cov_42.1717_ID_3, 4515‐4455
pLM5446	*Listeria* sRNA Rli28	RF01492	NODE_11_length_53770_cov_127.625_ID_2, 53283‐53095
	*Listeria* sRNA Rli28	RF01492	NODE_17_length_1354_cov_77.15_ID_2977, 729‐914
	NiCo riboswitch	RF02683	NODE_11_length_53770_cov_127.625_ID_2, 34040‐34148
	RNA anti‐toxin A (RatA)	RF01776	NODE_17_length_1354_cov_77.15_ID_2977, 1055‐968

**Figure 2:**
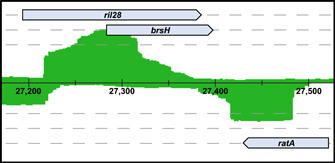
Genetic organization and read mapping of the *rli28*, *brsH*, and *ratA* genes of pLM80, pLM5446, and pLM7802. The *rli28*, *brsH*, and *ratA* loci of pLM80, pLM5446, and pLM7802 are identical; thus, the locus from pLM80 was chosen as the representative for the figure of the overall genetic organization. The numerical track is delimited in 100 bp segments and represents the location of the genes within the pLM80 sequence. Dashed gray lines represent different reading frames of the strands with those that are above the numerical track representing the positive strand and those below indicating the negative strand. Transcriptome reads (green) shown mapped to the pLM80 sequence in this figure were derived from Guariglia‐Oropeza et al. (2018) control samples of *Listeria monocytogenes* H7858 and display the areas and the degree of DNA transcription in relation to the predicted genes. Read mapping was performed with ReadXplorer (Hilker et al., 2016).

The second pLM80 ncRNA predicted is *ratA*. This is the first time *ratA* is found within the sequences of an *L. monocytogenes* plasmid. RatA is annotated as part of a TxpA/RatA type I toxin−antitoxin system first described in *Bacillus subtilis* (Rfam: RF01776) (Silvaggi et al., 2005). Type I toxin−antitoxin systems are characterized by a toxic protein component paired with an antisense RNA that binds to a region of the toxin mRNA, thereby preventing unwanted translation of the toxin (Nonin‐Lecomte et al., 2021; Yang & Walsh, 2017). Notably, *ratA* is flanked by *rli28* on the complementary strand (Figure 2).

While no homolog of *txpA* was identified on pLM80 (or pLM5446 and pLM7802, mentioned below), a gene encoding a predicted hypothetical protein (Locus_tag: LMOh7858_pLM80_0050) was found immediately downstream of *ratA* on the opposite strand and overlaps the *rli28* sequence. Comparing the putative protein's sequence against the UniProt database revealed a match with 38% amino acid identity and 77% coverage to BsrH, a probable toxin encoded in the genome of *B. subtilis*. In *B. subtilis*, *bsrH* is found immediately adjacent to the *txpA/ratA* toxin−antitoxin locus within the *B. subtilis* phage‐like region known as the *skin* element (48 kb in size), and it is thought to be part of a type I toxin−antitoxin cassette similar to TxpA/RatA (Irnov et al., 2010; Silvaggi et al., 2005). It is worth noting that BsrH was first identified as a lncRNA, and its role as a protein‐encoding mRNA was only later elucidated (Irnov et al., 2010). The putative pLM80 *bsrH* gene overlaps *rli28* by 105 bp on the same strand and extends 14 bp past the 3’ end of *rli28* (Figure 2). It is tempting to speculate that Rli28 may be a *cis‐acting* (ncRNAs that affect mRNA encoded in the same locus) signal‐response regulator for the putative toxin, as discussed briefly in other systems (Irnov et al., 2010), and that RatA may act as the antitoxin ncRNA for this system. However, this regulatory relationship needs to be experimentally verified in future studies.

### 3.2 Genetic analysis of pLM7802

We found that pLM7802 was assembled into 12 contigs with an assembly size of 135 kbp and contained two RepA proteins, one from Group 1 (Locus_tag: KJS00_15640) and the other from Group 2 (Locus_tag: KJS00_15040) (Table 3). The presence of two distinct RepA proteins may indicate that pLM7802 represents two plasmids, and indeed the phenomenon of *L. monocytogenes* strains harboring two plasmids has been reported before (Harrand et al., 2020; Hingston, Chen, Dhillon, et al., 2017; Kolstad et al., 1991; Korsak et al., 2019; Lebrun et al., 1992; Romanova et al., 2002). Alternatively, pLM7802 may comprise a single plasmid with two distinct *repA* genes, as previously shown for other *L. monocytogenes* plasmids such as pLMN1‐011A (Chmielowska et al., 2021; Korsak et al., 2019; Schmitz‐Esser et al., 2021). However, as pLM7802 is assembled into several contigs, there is no definitive evidence as to whether or not pLM7802 represents two individual plasmids or a singular, composite plasmid. As the presence of two plasmids in the same *L. monocytogenes* strain is considered rare (Chmielowska et al., 2021; Korsak et al., 2019; Schmitz‐Esser et al., 2021), we assumed that pLM7802 represents one plasmid.

Evaluating the pLM7802 curated annotations revealed several genes of interest, most of them related to stress tolerance. Within the pLM80‐like module of pLM7802 were genes encoding nearly identical (>99% amino acid identity to the pLM80 proteins) copies of CadA2C2 (Locus_tags: KJS00_15155; KJS00_15150), BcrABC (Locus_tags: KJS00_15105; KJS00_15100; and KJS00_15095), Tmr (Locus_tag: KJS00_15080), and a Group 2 RepA (Locus_tag: KJS00_15040). Four putative UvrX proteins are encoded on four separate small contigs (609−2441 bp in size) contained in pLM7802. However, they were significantly shorter than the full‐length UvrX and most likely represent pseudogenes caused by the fragmented assembly.

Other pLM7802 stress‐associated proteins of interest include a putative ATP‐dependent ClpB‐like protease (Locus_tag: KJS00_15045). ClpB proteins of other bacteria have important roles in virulence and stress response (particularly in heat stress) (Alam et al., 2021). The pLM7802‐encoded ClpB is identical to a previously identified ClpB found on the *L. monocytogenes* plasmid p4KSM (Naditz et al., 2019). Although neither of these plasmid‐encoded ClpB proteins has been functionally characterized, Naditz et al. (2019) proposed a potential role during heat stress. One pLM7802 gene encodes a putative heavy metal‐binding protein (Locus_tag: KJS00_15295), and three others (Locus_tags: KJS00_14715; KJS00_15300; and KJS00_15335) encode putative heavy metal translocating ATPases; however, these proteins have no functionally characterized homologs in *Listeria* or other bacteria. All four proteins have identical homologs on the plasmid pLM5446 (discussed below).

NADH peroxidases (Npr) are putative stress response elements commonly encoded on some *L. monocytogenes* plasmids (Hingston et al., 2019; Kuenne et al., 2010; Schmitz‐Esser et al., 2021). pLM7802 contains a putative *npr* (KJS00_14735) with a possible role in oxidative stress (Hingston et al., 2019).

Interestingly, pLM7802 encodes a much larger MCO (528 amino acids) than those of *S. aureus* and pLMR479a (386 amino acids) (Appendix Figure A1), and the pLM7802 MCO shares 40% amino acid identity and 62% sequence coverage with the MCO of pLMR479a.

pLM7802 contains a putative fluoride tolerance operon which includes a fluoride riboswitch and two predicted fluoride efflux transporter CrcB family proteins (Locus_tags: KJS00_15390 and KJS00_15395). Close homologs of these two proteins are found in many *Listeria, Clostridium*, and *Bacillus* species (Baker et al., 2012; Hingston et al., 2019; Stockbridge et al., 2012). The characterized *Escherichia coli* and *Bacillus cereus* CrcB proteins are regulated by fluoride and increase tolerance toward high fluoride concentrations (Baker et al., 2012). Fluoride exhibits antimicrobial activity in water (Marquis, 1995); thus, increased tolerance to fluoride supplemented in tap water may enhance the stress survival of *L. monocytogenes* in food and FPEs.

Finally, four putative ncRNAs were predicted to be encoded on the pLM7802 plasmid contigs (Table 4): the aforementioned fluoride riboswitch, identical copies of the pLM80 *ratA* and *rli28*, and a putative NiCo riboswitch previously described in the *L. monocytogenes* plasmid pLMR479a (Cortes et al., 2020). Regarding the pLM7802 *ratA*, pLM7802 also harbors the same putative toxin gene identified on pLM80 (Locus tag: LMOh7858_pLM80_0050).

### 3.3 Genetic analysis of pLM5446

pLM5446 was assembled into four contigs, has an assembly size of 73 kbp, and contains a Group 1 RepA protein gene (Locus_tag: KJR89_15050) (Table 3). Additionally, pLM5446 harbors several annotated stress‐associated genes, including *bcrABC* (Locus_tags: KJR89_14985, KJR89_14980, KJR89_14975), *cadA2* and *cadC2* (Locus_tags: KJR89_14955 and KJR89_14960), *npr* (Locus_tag: KJR89_15095), and an *mco* identical to the pLM7802 *mco* (Locus_tag: KJR89_15410). Similar to pLM7802, two genes encode proteins annotated as heavy metal‐binding proteins (Locus_tags: KJR89_15445 and KJR89_15405), and two others encode putative translocating ATPases (Locus_tags: KJR89_15110 and KJR89_15440). A putative UvrX protein encoded on pLM5446 shares 97% amino acid identity and 100% sequence coverage with the pLM80 UvrX. Also, a putative cadmium resistance transporter, CadD (Locus_tag: KJR89*_*15115), shares 43% amino acid identity and 86% sequence coverage with a functionally characterized CadD from *S. aureus* that increased tolerance to cadmium (Crupper et al., 1999). pLM5446 encodes a putative type II toxin−antitoxin system (Locus_tags: Doc toxin ‐ KJR89_15185; Phd antitoxin—KJR89_15190). This toxin cassette was found on many *L. monocytogenes* plasmids and may be involved in stress response or plasmid maintenance (Anast & Schmitz‐Esser, 2020; Kamruzzaman & Iredell, 2019; Kuenne et al., 2010). Finally, we identified four putative ncRNAs harbored within the pLM5446 plasmid contigs (Table 4): two copies of *rli28* found on separate contigs, *ratA*, and a putative NiCo riboswitch. Similar to pLM7802 and pLM80, pLM5446 harbors the same putative toxin component of the RatA toxin‐antitoxin system.

### 3.4 General gene expression characteristics of *L. monocytogenes* plasmids

After quality filtering of the transcriptome reads and mapping against the respective *L. monocytogenes* genomes, plasmid coverages ranged from 21× to 420×, and total read‐depth per sample (including chromosome and plasmid contigs) ranged from 1.6 to 38.8 million (Table 5 and Table S2: https://doi.org/10.6084/m9.figshare.20558475). Average percentages of reads mapped to plasmids were 0.9, 1.3, and 0.7 for pLM80, pLM7802, and pLM5546, respectively. This average plasmid read coverage is consistent with values from other studies (Anast & Schmitz‐Esser, 2020; Cortes et al., 2020; Kragh & Truelstrup Hansen, 2019). It should be noted that only significantly DE genes are reported throughout the article.

**Table 5:** Plasmid transcriptome sequencing read‐mapping statistics

Study sample type	Plasmid	Total mappings^a^, ^b^	Plasmid‐mapped reads^a^	Chromosome coverage^a^	Plasmid coverage^a^	Percent reads mapped to plasmid^a^
Tang et al. (2015)						
CSS	pLM80	12,292,778	34,349	424×	42×	0.28
Modified BHI broth	pLM80	6,056,046	38,269	208x	47×	0.85
Guariglia‐Oropeza et al. (2018)						
Bile exposed	pLM80	14,967,079	167,791	512×	204×	1.11
Control BHI	pLM80	14,009,046	195,304	478×	237×	1.43
Assisi et al. (2021)						
FSL R8 7802 biofilm	pLM7802	14,975,260	196,862	496×	145×	1.35
FSL R8 7802 planktonic	pLM7802	21,280,268	281,997	704×	207×	1.22
FSL R8 5446 biofilm	pLM5446	17,001,079	146,884	557×	201×	0.86
FSL R8 5446 planktonic	pLM5446	14,514,911	76,382	477×	104×	0.53

Abbreviations: BHI, brain heart infusion; CSS, cold‐smoked salmon.

^a^ Values are reported as averages of exact conditional replicates.

^b^ Total mappings denote total reads mapped to the entire genome, including chromosome and plasmid contigs.

### 3.5 pLM80 gene expression results

After *L. monocytogenes* strain H7858 was exposed to 1.1% porcine bile, the expression of 30 pLM80 genes (32% of total plasmid genes) significantly changed (Table S1: https://doi.org/10.6084/m9.figshare.20558421). Seventeen genes were upregulated, while 13 were downregulated with log_2_ fold changes (FC) ranging from −2.1 to 3.2. Most DE genes (*n* = 16) were annotated as proteins of unknown function. Notable genes that were upregulated in response to bile exposure included (1) the replication‐associated genes *repA* (Locus_tag: LMOh7858_pLM80_0093) and *parA* (Locus_tag: LMOh7858_pLM80_0092), which may indicate an increase in *L. monocytogenes* plasmid replication and cell division; (2) the ncRNA *rli28*, potentially revealing an increase in the cell's effort to maintain the plasmid, assuming that the internal putative toxin protein is translated; and (3) *cadA2* (Locus_tag: LMOh7858_pLM80_0083). Previously, acid and salt stress influenced the expression of *cadA2*, *repA*, and *parA* plasmid genes in other *L. monocytogenes* strains (Cortes et al., 2020; Hingston et al., 2019). Only two genes assigned with a putative function were downregulated: *tmr* (Locus_tag: LMOh7858_pLM80_0067) and a gene encoding a Type IV secretion system protein (Flp pilus assembly complex ATPase component, Locus_tag: LMOh7858_pLM80_0010).

TPMs of each experimental replicate were averaged by condition to form a consensus value and ranked by most to least expressed gene. In addition to differential gene expression, overall gene expression levels can also provide useful information. The highest expressed pLM80 genes from the Guariglia‐Oropeza et al. (2018) bile and control replicates comprised *rli28*, *ratA*, a gene encoding a hypothetical protein (LMOh7858_pLM80_0089), *tmr*, and a transposase (LMOh7858_pLM80_0052). Two genes of interest were highly expressed in only the bile‐exposed samples: a hypothetical protein (LMOh7858_pLM80_0048) and *repA*. In most cases, the highest expressed genes in both control and experimental replicates were consistently the same in all studies analyzed here. The high expression of the same genes in diverse conditions suggests that certain *L. monocytogenes* plasmid genes are important regardless of the environmental conditions present.

Comparing the transcriptomes of *L. monocytogenes* H7858 grown on vacuum‐packed CSS to cultivation in MBHIB revealed that the expression of 45 pLM80 genes (48% of total genes) was significantly different (37 upregulated and 8 downregulated with log_2_ FCs ranging from −1.2 to 6.8). The majority of DE genes (*n* = 30) were annotated as proteins of unknown function. Remarkably, all upregulated genes were found within the predicted transfer and plasmid maintenance region (approximately between locus_tags LMOh7858_pLM80_0005 and LMOh7858_pLM80_0049) that shows similarity to the *Bacillus* plasmids pXO2 and pWA63 (Korsak et al., 2019; Kuenne et al., 2010; Van Der Auwera et al., 2005). These data suggest that conjugation may occur on the surface of CSS. Indeed, two of these genes are annotated as encoding elements of a putative type IV secretion system, the Flp pilus assembly complex ATPase component (Locus_tag: LMOh7858_pLM80_0010), and a TraG/TraD family protein (Locus_tag: LMOh7858_pLM80_0022). The ability of pLM80 to transfer by conjugation is further supported by the fact that it is virtually identical to pLIS1, a plasmid from a *Listeria welshimeri* strain that showed high transformation efficiency from *L. welshimeri* to *L. monocytogenes* (Korsak et al., 2019). Overall, the data indicate that the conjugation of pLM80 during growth on CSS may occur.

In the same study, Tang et al. (2015) utilized the same batch of commercially produced wet‐cured CSS fillets as Kang et al. (2012) used to characterize the composition and physicochemical characteristics of the naturally occurring commensal bacteria found on the CSS fillets. Most of the CSS microbiota was comprised of lactic acid bacteria (LAB) found at concentrations as high as 7 log CFU/g (Kang et al., 2012). It has been established that LAB compete against and even inhibit *L. monocytogenes* proliferation and survival (Gómez et al., 2016; Lewus et al., 1991; Scatassa et al., 2017). Therefore, perhaps possession of pLM80, or other similar plasmids that harbor several genes advantageous in FPEs, may increase the competitive fitness of *L. monocytogenes* toward bacteria competing with it for resources. Indeed, in previous competition experiments, *L. monocytogenes* plasmid genes were DE when *L. monocytogenes* was cocultured with food production bacteria (Anast & Schmitz‐Esser, 2020). Therefore, the expression of plasmid‐encoded genes may have been influenced by other microorganisms found on the CSS fillets.

Plasmid genes of interest that were downregulated in the Tang et al. (2015) data set include *repA*, *parA*, *cadA2*, and *cadC2*. The downregulation of the plasmid maintenance genes *repA* and *parA* is interesting because it suggests that plasmid replication and partitioning mechanisms may occur less frequently on CSS than in MBHIB. Assessing pLM80 gene expression levels from Tang et al. (2015) samples revealed that the most expressed genes in the control (MBHIB) and experimental (CSS) replicates were within the same region that includes the *bcrABC* cassette, *tmr*, a gene encoding a putative glyoxalase family protein, and two putative resolvases (Table S3: https://doi.org/10.6084/m9.figshare.20558514).

Lastly, 13 pLM80 genes were DE in both studies included here (Guariglia‐Oropeza et al., 2018; Tang et al., 2015) and are listed in Table 6 with their corresponding log_2_ FCs. Except for one hypothetical protein, all genes that were DE in both studies showed opposite changes in expression in the two studies. Together with the differences in gene expression described above, this suggests that the pLM80 gene expression patterns are specific to the fundamentally different conditions applied in the two studies.

**Table 6:** pLM80 genes that were DE during growth on CSS and exposure to bile

pLM80 locus_tag	Product	Tang et al. (2015) log_2_ FC	Guariglia‐Oropeza et al. (2018) log_2_ FC
LMOh7858_pLM80_0004	Hypothetical protein	0.7	1.7
LMOh7858_pLM80_0008	Hypothetical protein (pXO2‐28)	4.5	−1.5
LMOh7858_pLM80_0009	Hypothetical protein	4.9	−1.9
LMOh7858_pLM80_0010	Flp pilus assembly complex ATPase component	4.6	−1.6
LMOh7858_pLM80_0011	Hypothetical protein	4.0	−2.1
LMOh7858_pLM80_0012	Hypothetical protein	5.3	−1.8
LMOh7858_pLM80_0016	Hypothetical protein	4.2	−2.0
LMOh7858_pLM80_0078	DNA transposition‐like protein	−1.0	0.6
LMOh7858_pLM80_0080	Site‐specific recombinase, resolvase family	−1.2	0.9
LMOh7858_pLM80_0081	Site‐specific recombinase, resolvase family	−1.1	0.9
LMOh7858_pLM80_0083	Cadmium‐transporting ATPase CadA2 (EC 3.6.3.3)	−0.7	0.7
LMOh7858_pLM80_0092	Replication‐associated protein/ParA family protein	−0.6	1.1
LMOh7858_pLM80_0093	RepA replication protein group 2	−0.4	2.5

Abbreviations: CSS, cold‐smoked salmon; DE, differentially expressed; FC, fold changes.

### 3.6 pLM7802 gene expression results

Analyzing the plasmid transcriptome of *L. monocytogenes* FSL R8‐7802 planktonically cultured in BHI broth compared to cells adhered to steel coupons revealed that 33 pLM7802 genes were DE (21% of genes on the plasmid). All DE genes were significantly upregulated with log_2_ FCs ranging from 1.1 to 3.8. Of these, 15 could not be assigned a predicted function. Three of the four loci comprising the putative UvrX protein gene (Locus_tags: KJS00_15680, KJS00_15805, and KJS00_15665) were DE with log_2_ FCs ranging from 1.9 to 3.2. It should be noted that the *uvrX* gene on pLM7802 has been assembled into four small contigs (see above). Other *L. monocytogenes uvrX*‐like plasmid genes have been DE during cocultivation with cheese bacteria, salt stress, and organic and inorganic acid stress (Anast & Schmitz‐Esser, 2020; Cortes et al., 2020; Hingston et al., 2019). Interestingly, putative *uvrX* genes were consistently DE in various *L. monocytogenes* stress exposure studies and during planktonic versus biofilm growth comparisons. These observations suggest that the putative *uvrX* gene has one or more functions advantageous for *L. monocytogenes* strains.

Next, two genes encoding putative heavy metal ATPases, KJS00_15300 (log_2_ FC 1.5) and KJS00_14715 (log_2_ FC 1.1), were upregulated (Tables S1: https://doi.org/10.6084/m9.figshare.20558421, Table S3: https://doi.org/10.6084/m9.figshare.20558475). Interestingly, while *cadC2* was upregulated in biofilm growth (KJS00_15150‐log_2_ FC 1.3), the associated *cadA2* gene (Locus tag: KJS00_15155) was not. Other notable annotated genes that were DE include KJS00_14990 (log_2_ FC 1.7), a putative Type IV secretion system component, and the NADH peroxidase gene KJS00_14735 (log_2_ FC 1.3). Prior research demonstrated that homologs of the gene encoding the putative NADH peroxidase in other *L. monocytogenes* plasmids were downregulated succeeding exposure to lactic acid stress and upregulated following exposure to inorganic acid and salt stress (Cortes et al., 2020; Hingston et al., 2019).

Additionally, two of the four predicted ncRNAs were significantly upregulated. The ncRNA *rli28* was the most upregulated pLM7802 gene (log_2_ FC 3.8), and the putative NiCo riboswitch was the seventh most upregulated (log_2_ FC 2.8). During acid stress, an identical copy of the pLM7802 putative NiCo riboswitch was highly upregulated on the plasmid pLMR479a (Cortes et al., 2020). The most expressed pLM5446 genes from both biofilm and planktonic replicates include *rli28*, the NiCo riboswitch, *npr*, *tmr*, a hypothetical protein‐encoding gene (KJS00_14815), and *bcrA* (Table S3: https://doi.org/10.6084/m9.figshare.20558514).

### 3.7 pLM5446 gene expression results

Comparing planktonic growth in BHI broth against *L. monocytogenes* FSL R8‐5446 in biofilm on stainless steel coupons revealed significant upregulation of 40 pLM5446 genes (51% of total plasmid genes) and significant downregulation of four others (Tables S1: https://doi.org/10.6084/m9.figshare.20558421 and Table S3: https://doi.org/10.6084/m9.figshare.20558514). The log_2_ FCs of these genes ranged from −2.2 to 5.2. Thirteen DE genes could not be assigned with a predicted function, and three were annotated as potential pseudogenes. Genes encoding the putative type II toxin−antitoxin system were upregulated (KJR89_15190—log_2_ FC 4.0; KJR89_15185—log_2_ FC 4.0); homologs of this system found on the plasmid pLM6179 were upregulated during coculture with cheese bacteria and exposure to lactic acid stress (Anast & Schmitz‐Esser, 2020; Cortes et al., 2020). As mentioned previously, pLM5446 encodes four putative genes involved in heavy metal resistance. All four genes were upregulated with log_2_ FCs ranging from 1.3 to 3.5 (Locus_tags: KJR89_15405, KJR89_15440, KJR89_15110, and KJR89_15445). One possible explanation for the significant upregulation of putative heavy metal tolerance genes is that cells adhered to stainless steel plates (ASI 304) in biofilm experiments. ASI 304 stainless steel contains iron, nickel, and chromium, which may leach from the coupons and come in contact with *L. monocytogenes* (Jellesen et al., 2006). In addition, other putative heavy metal tolerance genes were upregulated as well: *cadA2* (Locus_tag: KJR89_14955, log_2_ FC 2.6), *cadC2* (Locus tag: KJR89_14960, log_2_ FC 1.1), and *cadD* (Locus tag: KJR89_15115, log_2_ FC 0.8).

Other genes of interest that were upregulated included *uvrX* (Locus tag: KJR89_15035—log_2_ FC 3.5), and, similar to pLM7802, the gene encoding the putative NADH peroxidase (Locus_tag: KJR89_15095) was upregulated in biofilm with a log_2_ FC of 1.2. Notably, the most upregulated genes were the two copies of *rli28* with log_2_ FCs of 5.2 and 4.2. Lastly, the putative NiCo riboswitch significantly increased in expression by a log_2_ FC of 1.8. Genes with the highest expression levels based on TPM values in biofilm and planktonic growth conditions encompass *rli28*, NiCo riboswitch, *npr*, and both genes of the toxin‐antitoxin cassette (Table S3: https://doi.org/10.6084/m9.figshare.20558514).

Comparing the differential gene expression of the pLM7802 and pLM5446 under the same conditions revealed that 11 shared genes were DE in both plasmids during growth in biofilm (Table 7). However, the majority of DE genes for each of the plasmids were plasmid‐specific. Interestingly, all shared DE genes were consistently upregulated during growth in biofilm. It is currently unknown which of these shared plasmid genes are involved in biofilm formation or if unique genes on individual plasmids might be involved in biofilm formation. Future studies will be needed to determine if any plasmid genes are involved in biofilm formation.

**Table 7:** Shared genes of pLM7802 and pLM5446 that were DE during growth in biofilm

pLM7802 locus_tag	pLM5446 locus_tag	Product	pLM7802 log_2_ FC	pLM5446 log_2_ FC
KJS00_15320	KJR89_15420	HAMP domain‐containing histidine kinase	2.5	2.6
KJS00_15455	KJR89_15140	Hypothetical protein	2.3	2.2
KJS00_15480	KJR89_15080	Hypothetical protein	2.1	1.1
KJS00_15145	KJR89_15010	Site‐specific recombinase, resolvase family	2.1	1.5
KJS00_15325	KJR89_15415	Two‐component transcriptional response regulator	2.1	2.3
KJS00_15475	KJR89_15085	Hypothetical protein	2.0	1.0
KJS00_15460	KJR89_15135	Hypothetical protein	1.8	1.9
KJS00_15300	KJR89_15440	Putative copper‐translocating P‐type ATPase CopA	1.5	2.3
KJS00_14735	KJR89_15095	NADH peroxidase Npr	1.3	1.2
KJS00_15150	KJR89_14960	Cadmium efflux system accessory protein CadC2	1.3	1.1
KJS00_14715	KJR89_15110	Lead, cadmium, zinc, and mercury transporting ATPase	1.1	1.4

Abbreviations: DE, differentially expressed; FC, fold changes.

### 3.8 Plasmid‐encoded ncRNAs exhibit high levels of transcription

Previous transcriptomic analysis of *L. monocytogenes* revealed notably high expression of chromosomal (Anast & Schmitz‐Esser, 2020; Cortes et al., 2020; Duru et al., 2021) and plasmid (Cortes et al., 2020) ncRNAs compared to the expression of other genes in the same conditions. Thus, in addition to elucidating differential expression of protein‐coding genes harbored on pLM80, pLM5446, and pLM7802, we sought also to determine if the putative ncRNAs described in this study were highly expressed relative to chromosomal and plasmid‐encoded protein‐coding genes (Table S3: https://doi.org/10.6084/m9.figshare.20558514). Remarkably, *rli28* and the putative NiCo riboswitch were among the five most expressed plasmid genes in the experimental and control replicates of most data sets described here, with the notable exception of *rli28* showing slightly lower TPM rankings of 9th and 11th in the replicates of Tang et al. (2015). The high expression levels of these ncRNAs indicate that plasmid ncRNAs may have a role in plasmid gene regulation and potentially regulate plasmid stress tolerance mechanisms. However, the function of these putative plasmid ncRNAs will need to be determined experimentally in future studies.

## 4 CONCLUSION

Until now, only three studies have analyzed the complete plasmid transcriptome profiles of *L. monocytogenes* strains (Anast & Schmitz‐Esser, 2020; Cortes et al., 2020; Kragh & Truelstrup Hansen, 2019). In the data sets reanalyzed here, we observed that many plasmid genes were DE (21%−51% of the total plasmid genes). Furthermore, a number of the aforementioned DE genes were also DE in previous analyses (Anast & Schmitz‐Esser, 2020; Cortes et al., 2020). Although most DE plasmid genes from this study possess no predicted function, the substantial number of DE genes in each data set suggests that plasmid genes have an important—yet unknown—role in the *L. monocytogenes* response to the tested conditions. Therefore, we emphasize the need for further analysis of these genes and their encoding plasmids to determine their functions and potential roles in the survival and persistence of *L. monocytogenes*.

Notably, even though *L. monocytogenes* strains FSL R8‐7802 and FSL R8‐5446 were grown under the same conditions (biofilm and planktonic growth), the gene expression patterns of their plasmids, pLM7802 and pLM5446, were highly distinct. These data suggest that even if plasmids harbor several genes encoding near‐identical proteins, gene expression seems to be plasmid‐specific even under the same experimental conditions. The difference in the expression of similar plasmid genes encoded on different plasmids may be due to divergent gene regulatory systems, plasmid‐ or chromosomally encoded. Plasmid−chromosomal cross‐talk of gene regulation systems does occur in other bacteria (Diel et al., 2019; Gong et al., 2013; Vial & Hommais, 2020) and may occur in *L. monocytogenes* as well.

We identified putative ncRNAs that were DE in multiple highly distinct conditions on every plasmid analyzed. Furthermore, *rli28* and the putative NiCo riboswitch were among the highest expressed genes in most *L. monocytogenes* plasmid transcriptomes across various conditions. These data strongly suggest a vital function, possibly in regulating stress response genes, of the plasmid‐encoded ncRNAs of *L. monocytogenes* and warrant further study into their possible implications in the persistence of *Listeria*.

## AUTHOR CONTRIBUTIONS

**Justin M. Anast**: Conceptualization (equal); formal analysis (lead); visualization (lead); writing–original draft (lead); writing–review and editing (equal). **Andrea J. Etter**: Resources (supporting); writing–original draft (supporting); writing–review and editing (supporting). **Stephan Schmitz‐Esser**: Conceptualization (equal); funding acquisition (lead); supervision (lead); writing–original draft (equal); writing–review and editing (equal).

## CONFLICT OF INTEREST

None declared.

## ETHICS STATEMENT

None required.

## Data Availability

The data sets analyzed during the current study are available at NCBI under the following BioProjects: The transcriptome sequencing data for pLM80 can be found under the following links: https://www.ncbi.nlm.nih.gov/bioproject/PRJNA270808 and https://www.ncbi.nlm.nih.gov/bioproject/PRJNA401799. The transcriptome sequencing data for pLM7802 and pLM5446 can be found at https://www.ncbi.nlm.nih.gov/bioproject/PRJNA736746. The FSL‐R8‐7802 whole genome shotgun sequencing data have been deposited at DDBJ/ENA/GenBank under the accession number JAGYVE000000000 and the FSL‐R8‐5446 whole‐genome shotgun sequencing data have been deposited at DDBJ/ENA/GenBank under the accession number JAGYVF000000000: https://www.ncbi.nlm.nih.gov/nuccore/JAGYVF000000000. **Table S1** is available at https://doi.org/10.6084/m9.figshare.20558421 (Table S1. Comparative genomics, proteomics, and transcriptomics of pLM80, pLM5446, and pLM7802. *L. monocytogenes* plasmid results are presented from left to right as follows: pLM5446, pLM7802, and pLM80 genome information and gene expression results from the studies by Assisi et al., 2021, Guariglia‐Oropeza et al., 2018, and Tang et al., 2015.) This is then followed by comparative protein blasts of pLM7802 against pLM80, pLM7802 against pLM5446, and pLM80 against pLM5446. The similarity between protein sequences is denoted as the percent amino acid identity shared between the plasmids and then by percent query coverage. Gray rows indicate that the corresponding CDS was duplicated within the plasmid module to align homologs found between each plasmid. This was done to account for several predicted protein sequences from pLM80 and pLM5446 that had multiple homologs within the pLM7802 proteome. Within the individual plasmid modules, “Locus tag” indicates the assigned locus tag of each predicted CDS, “Product” denotes the predicted final product from each gene, “Gene name” designates the short name of each gene where applicable, and “Pfam protein domain(s)” shows the predicted Pfam protein domains within each amino acid sequence generated by the eggNOG‐mapper (http://eggnog-mapper.embl.de/). Gene expression results are first assigned by the publication, followed by either “log2FoldChange” (log_2_ fold change of gene) or “Q‐value” (*p* value adjusted for errors from multiple testing). Gene expression data are only displayed for genes that had *Q* values ≤ 0.05; **Table S2** is available at https://doi.org/10.6084/m9.figshare.20558475 (Table S2. Mapping read statistics of individual replicates from the reanalysis of plasmid data of Assisi et al., 2021, Guariglia‐Oropeza et al., 2018, and Tang et al., 2015.) Data are presented by study, from top to bottom: (1) Tang et al. (2015), (2) Guariglia‐Oropeza et al. (2018), and (3) Assisi et al. (2021). “Sample” specifies from what sample replicate each statistic data are generated. The “SRA accession” column lists the sequence read archive (SRA) accession number for the corresponding sample. It is important to note that reads from the Guariglia‐Oropeza et al. (2018) study that were submitted to the SRA are not the raw reads containing pLM80 reads. We obtained unfiltered reads by directly contacting the authors of this study. “Plasmid” denotes what plasmid is harbored within the strain from the sample. “Total mappings” means the total reads that were mapped to the chromosome and plasmid contigs. “Length of the entire genome (sum contigs)” shows the sum of all base pairs from the chromosome and plasmid contigs. “Length of plasmid (sum contigs)” designates the sum of base pairs of the predicted plasmid contigs. “Reads mapped to plasmid” displays the total reads mapped to plasmid‐only contigs. “Chromosome coverage” denotes the total read coverage of all the chromosome‐only contigs. “Plasmid coverage” indicates the total read coverage of all plasmid‐only contigs. “Percent of Total mappings that are attributed to plasmid contigs” shows the percentage of all mapped reads in the sample that were assigned to plasmid contigs; **Table S3** is available at https://doi.org/10.6084/m9.figshare.20558514 (Table S3. Normalized gene expression levels of genes from the plasmids pLM7802, pLM5446, and pLM80.) Results are separated by plasmids in sheets named after the plasmids pLM7802, pLM5446, and pLM80. At the highest level, results are presented by study, then by experimental condition, and lastly, by expression results and locus tags or ncRNA name. “Locus tags/ncRNA name” indicates the assigned locus tag of each predicted CDS or the name of a predicted ncRNA. “TPM” denotes the transcript per million value for each gene generated by ReadXplorer and with TPM values of each replicate averaged together in R. “Rank” shows the numerical order from greatest TPM value to smallest TPM value starting with the greatest value as “1” relative to the plasmid genes of each experimental condition. A TPM value of “‐” indicates that no reads were mapped for that specific gene in the corresponding data set).

## APPENDIX A

See Figure A1 and Table A1.

**Table A1:** Pairwise average nucleotide identities of plasmids pLM7802, pLM80, and pLM5446.

	pLM7802	pLM80	pLM5446
pLM7802		99.98 [61.62]	98.81 [39.39]
pLM80	99.98 [99.26]		98.80 [14.60]
pLM5446	98.81 [71.34]	98.80 [18.09]	

*Note*: Percent similarity was calculated with the JSpecies webserver (http://jspecies.ribohost.com/jspeciesws/) mummer algorithm. The average nucleotide identity is shown as the percent identity, and alignment coverage is shown as a percentage within brackets.
